# Multiple Myeloma Treatment Challenges: A Case Report of Vertebral Artery Pseudoaneurysm Complicating Occipitocervical Arthrodesis and a Review of the Literature

**DOI:** 10.7759/cureus.49716

**Published:** 2023-11-30

**Authors:** Gervith Reyes Soto, Carlos Salvador Ovalle Torres, Jorge Perez Terrazas, Kaori Honda Partida, Andreina Rosario Rosario, Alvaro Campero, Matias Baldoncini, Manuel de Jesus Encarnacion Ramirez, Nicola Montemurro

**Affiliations:** 1 Neuroscience Unit, Instituto Nacional de Ciencias Médicas y Nutrición Salvador Zubirán, Mexico City, MEX; 2 Neurosurgery, National Autonomous University of Mexico (UNAM) Hospital General de Mexico, Mexico City, MEX; 3 Spine Surgery, National Autonomous University of Mexico (UNAM) Hospital General de Mexico, Mexico City, MEX; 4 Medical School, Autonomous University of Santo Domingo (UASD), Santo Domingo, DOM; 5 Neurological Surgery, Hospital Padilla, Tucumán, ARG; 6 Neurosurgery, School of Medicine, University of Buenos Aires, Buenos Aires, ARG; 7 Neurosurgery, San Fernando Hospital, Buenos Aires, ARG; 8 Neurological Surgery, Peoples Friendship University of Russia, Moscow, RUS; 9 Neurosurgery, Azienda Ospedaliera Universitaria Pisana (AOUP) University of Pisa, Pisa, ITA

**Keywords:** spine oncology, multiple myeloma, arthrodesis, endovascular treatment, surgical complication, cervical spine

## Abstract

Multiple myeloma is a hematological neoplasm that frequently affects the spinal column. Less than a fifth of this vertebral involvement corresponds to the cervical spine and cranio-cervical junction. When there is instability or neurological involvement due to compression or deformity, approaches for anterior decompression and occipitocervical stabilization are required. The correct managment of vertebral artery aneurysm associated with occipitocervical arthrodesis requires extensive knowledge of anatomy and pathology. We present a case of a vertebral pseudoaneurysm that occurred late after the resection of a C1-C2 vertebral body multiple myeloma lesion managed with endonasal endoscopic approach and posterior occipitocervical arthrodesis as well as a systematic review of the related literature. The patient recovered well, without major neurological deficits.

## Introduction

Multiple myeloma (MM) is the second-most common hematological malignancy in adults, accounting for up to 1.8% of all cancer cases [1]. Vertebral involvement secondary to this neoplasm is common because the vertebrae contain many hematopoietic cells in their bone marrow. MM spinal metastases frequently occur in the cervical segment and often can cause instability or compression at the cranio-cervical junction with significant neurological involvement [2], which requires complex approaches for correction and treatment and often requires anterior, posterior, or combined approaches to achieve effective neurological decompression and subsequent stabilization [3]. The key to decision-making is evaluating and monitoring the biomechanical stability of the spine as part of a multidisciplinary approach [4,5], considering that surgery should be considered for cases of spinal instability and potential neurological injury [6]. Traditionally, the transoral route has been favored for anterior decompression of the cranio-cervical junction. However, in recent decades, the endonasal endoscopic route has gained acceptance as a surgical corridor for the treatment of congenital or acquired cranio-cervical junction (CCJ) pathology because it allows for effective neural decompression, improving the deficit in most affected patients, including MM metastases, in addition to the advent of advances in stereotactic radiosurgery, encompassing the neurological, oncological, mechanical, and systemic (NOMS) decision framework [7-11]. The posterior approach to the cervical spine is a common surgery with few complications; occipitocervical arthrodesis is indicated when the CCJ instability occurs. Known risks from the posterior spine approach are due to complex regional vascular neural anatomy and may generate neurological or vascular complications, especially those associated with early or late vertebral artery injury, either of which can be fatal if not quickly recognized. Therefore, it is crucial to prevent this complication and know how to investigate it upon suspicion of vertebral artery (VA) injury [12-16]. Imaging studies play a fundamental role in determining the course of treatment [17]. Another critical aspect is the anatomy of the vertebrobasilar system; the anatomical variants of the VA must be known, as their presence may increase the risk of iatrogenic injury to the VA [18].

## Case presentation

Clinical case

**Figure 1:**
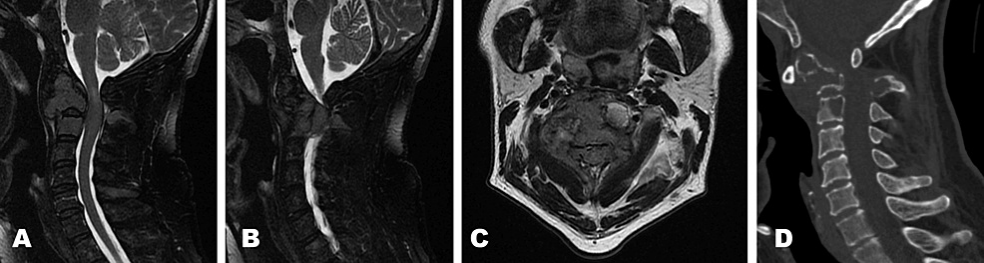
Sagittal (A, B) and axial (C) T2 sequence MRI shows vertebral bodies with heterogeneous signal and uptake throughout the entire spine secondary to multiple myeloma, with greater involvement at levels C1, C2, and C3. In this last segment, the infiltrative tissue involves adjacent paravertebral tissues and extends into the spinal canal, causing a narrow canal and compressing the adjacent spinal cord (C), which shows an increased signal, findings consistent with compression myelopathy. There is involvement of foramina and nerve roots bilaterally at levels C1-C2 and C2-C3. It presents as an osteodestructive lesion in the CT scan (D).

A 48-year-old male patient was diagnosed with kappa-positive multiple myeloma, confirmed by histopathology following a biopsy. Upon neurological examination, he presented with cervical pain and paresthesias in the thoracic extremities, as well as reduced strength (4/5) and bilateral hyperreflexia. Magnetic resonance imaging (MRI) was performed, revealing a C2 fracture (Figure 1). 

**Figure 2:**
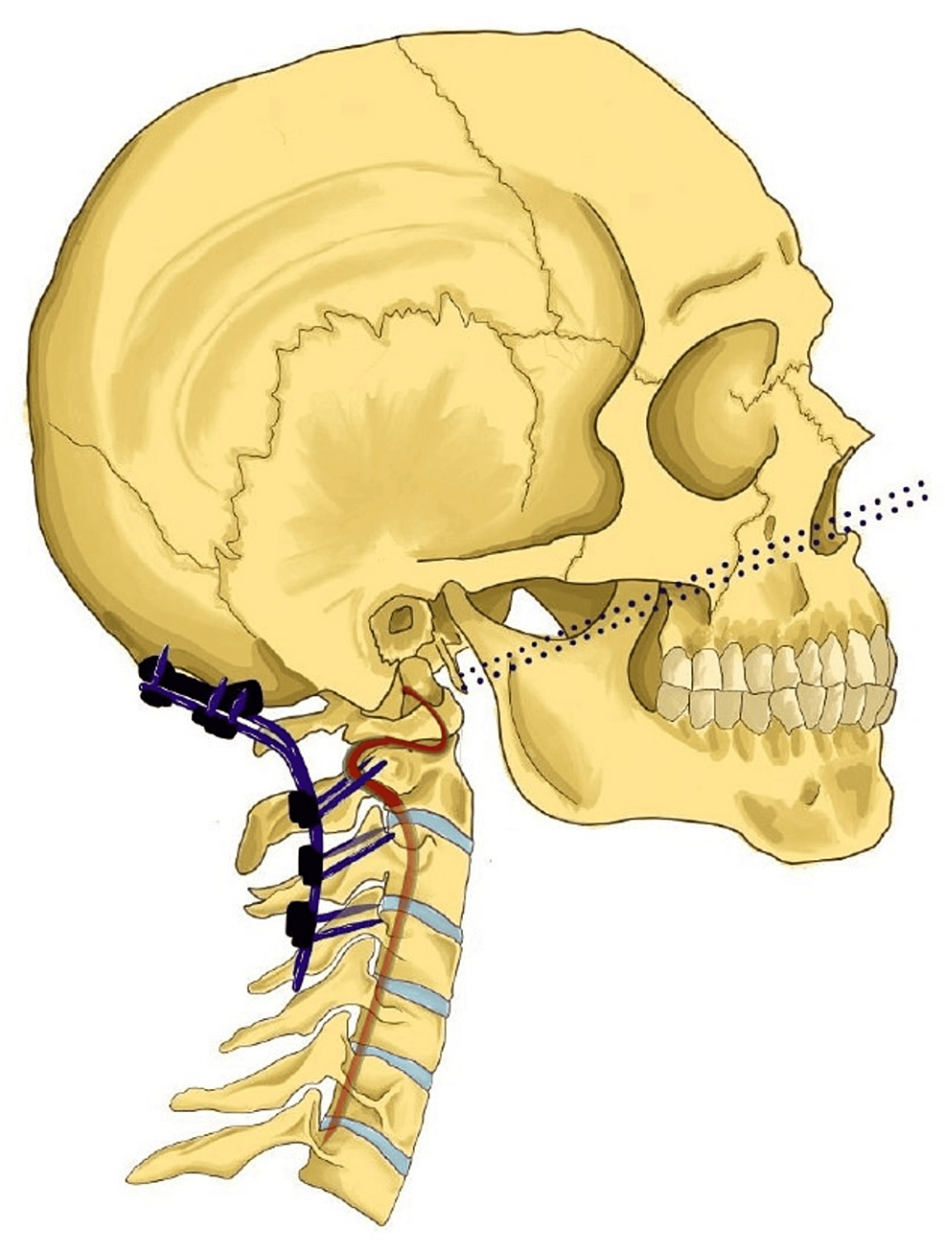
Representative Illustration of the main approaches in this case; via posterior, the occipitocervical fixation and screws can be seen (red line) and via anterior the trajectory of the endonasal endoscopic approach to the odontoid process is visible (dotted lines), as well as their relationship with the vertebral artery in the sagittal plane view. Figure owned by Manuel Encarnacion Ramirez.

He initially received radiotherapy treatment of 30 Gy in 10 fractions due to spinal compression. After cycles of radiotherapy, he continued to exhibit neurological symptoms and even showed a decrease in strength and sensitivity in the thoracic limbs. It was then decided to perform an odontoidectomy and endoscopic endonasal decompression, followed by posterior stabilization with occipitocervical arthrodesis (Figure 2).

**Figure 3:**
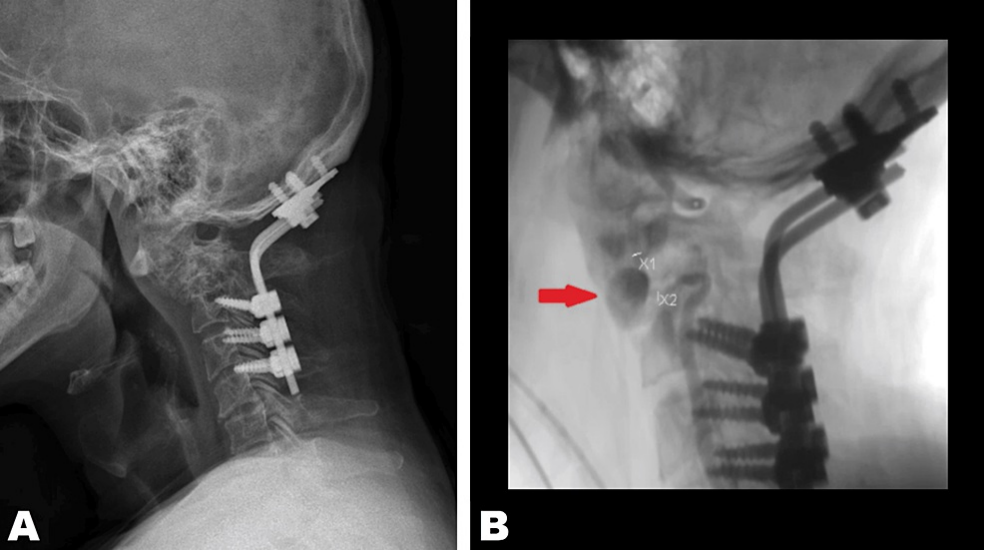
(A) Postoperative X-ray shows occipitocervical arthrodesis. (B) DSA shows a pseudoaneurysm in the V3 segment of the left vertebral artery (red arrow). DSA: digital subtraction angiography

**Figure 4:**
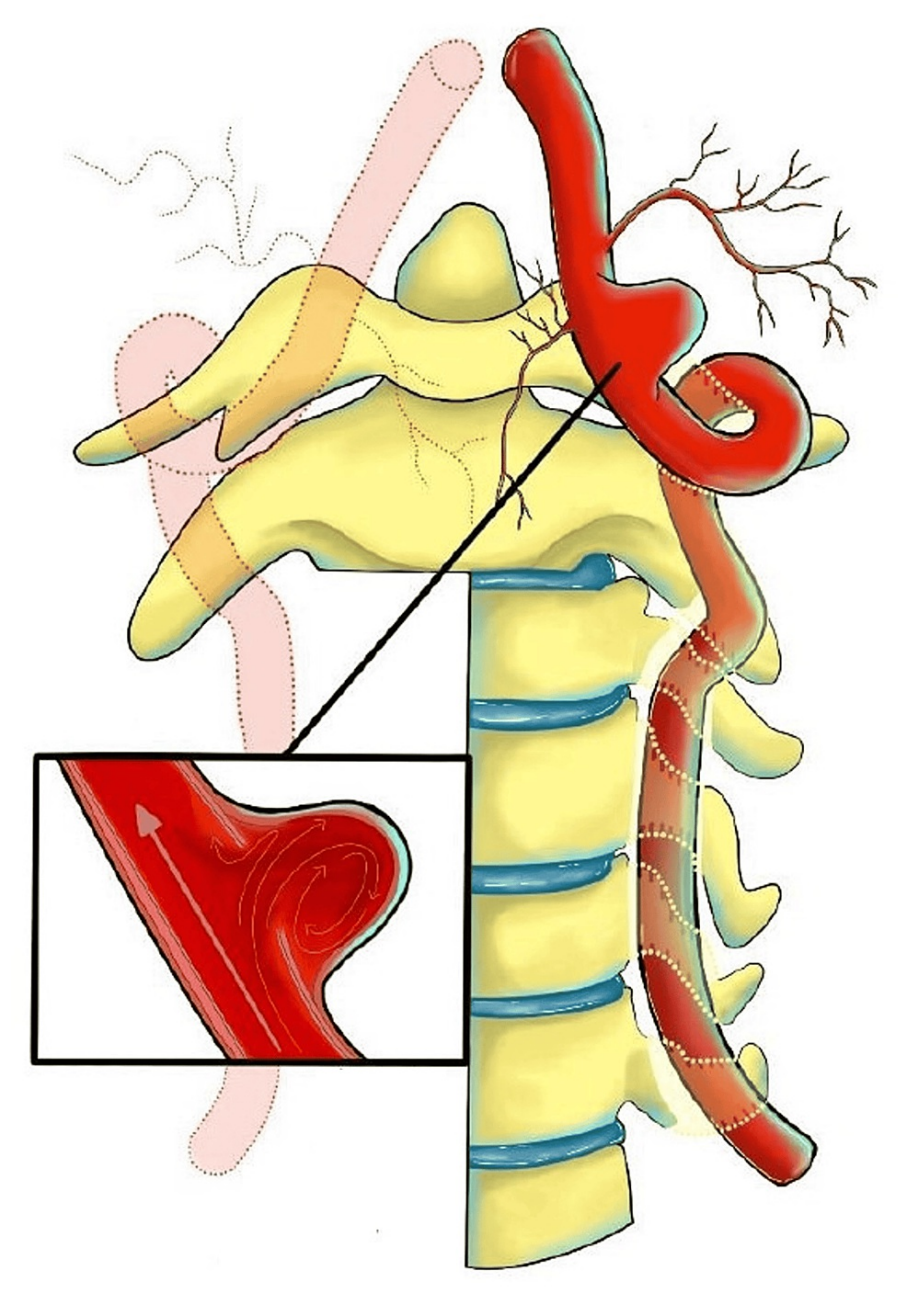
This illustration depicts the characteristics and localization of the vertebral artery pseudoaneurysm located between the segments V3; also the dilatation of the vessel is illustrated (inset image), including two layers of the vessel as injured layers (intima and media) with the consequent dilatation only of the adventitia layer, forming the pseudoaneurysm, causing a volumetric effect on the surrounding tissues, as well as its turbulent flow content. Figure owned by Manuel Encarnacion Ramirez.

Both procedures were performed without complications, and the patient showed improvement in motor and sensory deficits. Two years after the procedure, the patient began clinically deteriorating with a syncopal episode. The patient underwent angiography of the external carotids and two vertebrae, revealing a pseudoaneurysm dependent on the V3 segment of the left vertebral artery (Figures 3-4). Endovascular occlusion of the left vertebral artery was performed during the same procedure, placing a covered stent without complications at short- and long-term follow-up.

Literature review

A comprehensive literature search was conducted using PubMed, Scopus, and Google Scholar databases. The literature search was performed using a combination of terms and keywords such as "multiple myeloma," "vertebral artery," "pseudoaneurysm," "occipitocervical arthrodesis," "endovascular treatment," and "cervical spine."

We included all original research articles, case reports, and case series that discussed vertebral artery pseudoaneurysm in relation to occipitocervical arthrodesis and multiple myeloma. Only studies published in English were included. Reviews, commentaries, editorials, clinical trials, and non-English articles were excluded.

**Figure 5:**
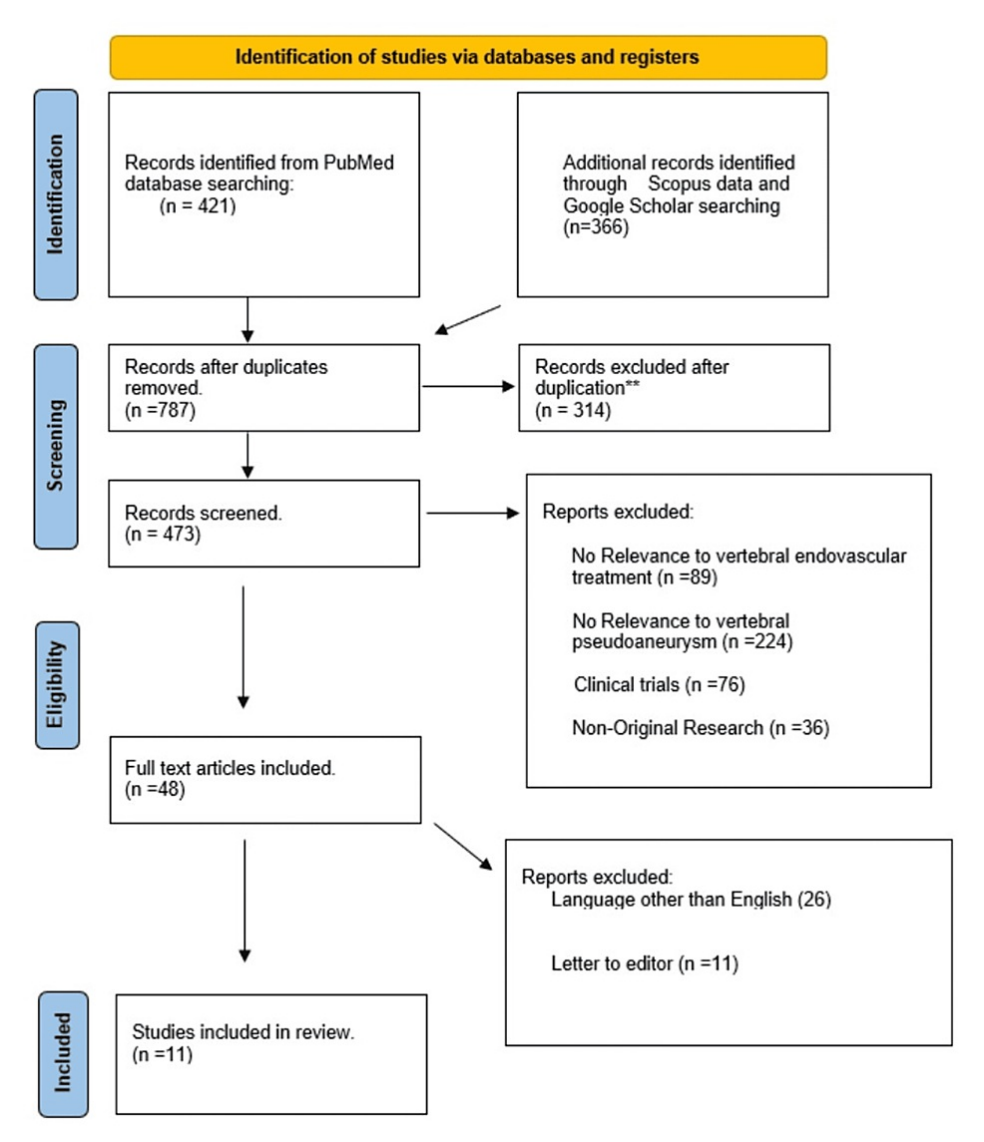
PRISMA flow diagram.

A systematic literature search initially surfaced 789 pertinent articles. However, after the diligent removal of duplicate entries and subsequent scrupulous screening of titles and abstracts, only 473 articles were deemed fit for a more comprehensive assessment for eligibility. After the first screening, 48 articles were selected and 425 were excluded. In the last screened series of articles, 11 were selected [15, 19-28]. Each of these 11 carefully assessed articles successfully met the predetermined inclusion criteria and was therefore integrated into the review, as delineated in (Tables 1-2). A detailed representation of the methodological progression and selection stages of the study is portrayed in the PRISMA flow diagram (Figure 5).

**Table 1:** Case reports on iatrogenic vertebral artery complications IP: intracranial pseudoaneurysm; CVC: central venous catheterization; VAVF: vertebral arteriovenous fistula; CIVAVF: CVCinduced VAVF; BCV: brachiocephalic vein; VA: vertebral artery

Title	Authors (Year)	Sample size	Methodology	Primary outcome
Vertebral artery dissection and associated ruptured intracranial pseudoaneurysm successfully treated with coil assisted flow diversion: A case report and review of the literature	Scullen et al. [19] (2021)	1	Single case report	The immediacy of treatment, the early exclusion of the ruptured IP, and the reconstruction and preservation of the diseased vessel contributed to a successful outcome
Vertebral artery transection with pseudoaneurysm and arteriovenous fistula requiring antegrade and retrograde embolization	Karatela et al. [20] (2022)	1	Single case report	Endovascular approaches have been more successful and have lower morbidity compared with conservative treatment or open surgery
Iatrogenic left vertebral artery pseudoaneurysm treated with a covered stent	Carrillo-Martínez et al. [21] (2020)	1	Single case report	Endovascular covered stent placement is an effective treatment method for the traumatically injured vertebral artery
Endovascular management of iatrogenic vertebral artery pseudoaneurysm: a case report	Park et al. [22] (2023)	1	Single case report	Good clinical outcomes
Iatrogenic vertebral arteriovenous fistula involving brachiocephalic vein due to central venous catheterization: a case report	Yamamoto et al. [23] (2022)	1	Single case report	Endovascular treatment may be feasible and useful for CIVAVF involving BCV
Self-expanding covered stent placement to treat a pseudoaneurysm caused by iatrogenic vertebral artery injury	Gigliotti et al. [24] (2021)	1	Single case report	There was good improvement in her cervical lordosis after her initial operation
Acute management of iatrogenic injury to vertebral artery with central venous catheter in a critically patient	Al Rayes et al. [25] (2020)	1	Single case report	The overall favorable outcome with a success rate of 85% to 89% and a reduction in the rate of perioperative complications
Acute endovascular therapy for iatrogenic vertebral artery injury: a case report	Ito et al. [26] (2022)	1	Single case report	Stent grafting led to a favorable outcome
Iatrogenic vertebral artery injury during anterior cervical spine surgery: a systematic review	Guan et al. [27] (2017)		Single case report	VA repair and stent placement had excellent outcomes
Delayed hemorrhage from an iatrogenic vertebral artery injury during anterior cervical discectomy and successful endovascular treatment—report of a rare case and literature review	Lo et al. [28] (2017)	1	Single case report	Endovascular embolization can be a safe treatment alternative to surgical repair

**Table 2:** Iatrogenic vertebral artery injury complications and recommendations DAPT: dual antiplatelet therapy; VAI: vertebral artery injury; PSA: pseudoaneurysm; AVF: arteriovenous fistula; EVD: external ventricular drain; VPS: ventriculoperitoneal shunt; DSA: digital subtraction angiography

Title	Author	Publication date	Technique used	Major complications	Recommendations
Endovascular management of iatrogenic vertebral artery pseudoaneurysm: a case report	Park et al. [22]	2023 Feb 8	Using multiple overlapping stents at the lesion site and microcatheter.	There were no complications, there were good clinical results after two years of follow-up.	Careful selection and observation of patients is recommended. Patients should receive DAPT to prevent thromboembolic complications.
Epidemiology and management of iatrogenic vertebral artery injury associated with cervical spine surgery	Yi et al. [15]	2022 Apr 19	Techniques to manage VAI include hemostatic tamponade, ligation, microvascular repair or anastomosis, and endovascular management.	An anterior approach can have complications such as dysphagia, esophageal injury, superior and recurrent laryngeal nerve palsies, and Horner's syndrome.	Immediate conventional angiography is recommended, followed by serial endovascular treatment and close follow-up of the patient.
Vertebral artery transection with pseudoaneurysm and arteriovenous fistula requiring antegrade and retrograde embolization	Karatela et al. [20]	2022 Mar 3	A 5F catheter was used in the distal left V2 segment and a combination of Headway 17 microcatheter (Terumo Corp, Tokyo, Japan) and Synchro-2 microwire (Stryker Neurovascular, Fremont, CA) was used up the basilar artery and down the right vertebral artery adjacent to the PSA and AVF.	Partial palsy of the right external oculomotor nerve and absence of light reflexes in the right cornea suggestive of brainstem ischemia.	Retrograde vertebral access can be especially useful for polytrauma patients who are too unstable for time-consuming, complex, open surgery.
Vertebral artery dissection and associated ruptured intracranial pseudoaneurysm successfully treated with coil assisted flow diversion: a case report and review of the literature	Scullen et al. [19]	2021 Jul-Sep	EVD was placed immediately on admission and before the intervention to avoid placing the catheter during therapeutic DAPT.	The patient had an uneventful postoperative course but required a VPS on postoperative Day 13.	Surveillance DSA should be used to visualize pseudoaneurysm growth and prevent catastrophic rupture.
Acute endovascular therapy for iatrogenic vertebral artery injury: a case report	Ito et al. [26]	2021 Apr 15	The 8-Fr sheath was inserted and endovascular therapy was performed.	There was no recurrence or ischemic complication.	Prevention of distal embolism and accurate hemostasis.
Self-expanding covered stent placement to treat a pseudoaneurysm caused by iatrogenic vertebral artery injury	Gigliotti et al. [24]	2021 Aug 13	Endovascular self-expanding covered stent.	There were no thromboembolic complications.	The choice of ticagrelor alone in this case demonstrates the potential benefit of utilizing this agent in preventing thromboembolic complications while minimizing the risk of hemorrhage.
Acute management of iatrogenic injury to the vertebral artery with a central venous catheter in a critically ill patient	Al Rayes et al. [25]	2020 Aug 23	Endovascular management was done and a 5-Fr sheath was placed.	No complications noted.	Endovascular management is minimally invasive and effective.
Iatrogenic left vertebral artery pseudoaneurysm treated with a covered stent	Carrillo-Martínez et al. [21]	2020 Sep 29	Endovascular covered stents.	Complications that can arise include rupture and thrombosis.	Vertebral artery pseudoaneurysm must be diagnosed and treated immediately because of its risk of rupture or thrombosis.

## Discussion

Vertebral pathology secondary to multiple myeloma is relatively common. One of the sites frequently affected is the cranio-cervical junction, which, due to its biomechanical complexity, requires an individualized and often combined approach to mitigate the patient's neurological deficit [29, 30]. Currently, it is recommended to perform an endoscopic endonasal odontoidectomy to achieve decompression and improve the patient's symptoms. It has been observed that vertebral artery injury from posterior spine surgery is a relatively uncommon but not rare complication. In a systematic review conducted by Yee and colleagues, they found from studies to date with a sample size of 3,884 patients that the incidence ranged from 0.2-2.2%, with a cumulative incidence of 0.4% [29]. This risk increases during C1-C2 arthrodesis, constituting up to 8.2% of injuries and 0.5% in anterior cervical treatment by decompression [12]. However, iatrogenic aneurysm associated with surgery is an exceptional event. It can generally develop from an arterial injury whose hemorrhage is controlled by tamponade and local hemostatic agents during surgery. It can also be caused by inadvertent trauma to the arterial wall during the surgical procedure. Currently, isolated cases without identifying the type of injury have been reported in the literature, focusing only on the location of the lesion [30-32]. They have been managed with the placement of an endovascular stent as in the present case. However, there is also the option of management with the placement of coils or their combined use [22]. There is also the possibility of management through endovascular embolization. Still, there are only reports of this management in pseudoaneurysms that occurred early on [33]. They generally appear secondarily, can go unnoticed, and sometimes manifest as cervical masses, the onset of tinnitus, or paralysis of cranial nerves [12].

During the anterior endonasal surgical approach, care must be taken to identify the midline in order to avoid vertebral artery injuries [34, 35]. That's why the longus colli muscles are significant reference points, as the lateral edges of the vertebral body are important markers [36, 37]. There is a clear dominance of endovascular interventions in the management of vertebral artery injuries. The shift towards these methods over traditional open surgeries may be attributed to their minimally invasive nature, potential for quicker recovery, and reduced risk of complications. As highlighted by Karatela et al. [20] and several others, endovascular approaches frequently result in positive outcomes with reduced morbidity compared to more invasive methods.

The majority of the presented cases report favorable outcomes following endovascular intervention. As seen in Park et al. [22] and Gigliotti et al. [24], there were no thromboembolic complications post-intervention. Such outcomes emphasize the safety and efficacy of these techniques, especially when employed promptly and with precision. The few complications reported, such as the need for a ventriculoperitoneal shunt by Scullen et al. [19], were generally manageable and didn't negate the benefits of the endovascular approach. Several studies emphasized the criticality of immediate diagnosis and intervention. Carrillo-Martínez et al. [21] highlighted the urgency of treating vertebral artery pseudoaneurysms due to their risk of rupture. This sentiment is further echoed in the recommendations of Yi et al. [15], which underscores the need for immediate angiography and thus reinforces the principle that timely action can prevent catastrophic outcomes.

Dual antiplatelet therapy after endovascular treatment appears to be a consistent recommendation to prevent thromboembolic complications [22]. Furthermore, the choice of specific antiplatelet agents, such as ticagrelor as reported by Gigliotti et al. [24], can offer benefits in minimizing risks of hemorrhage while preventing thromboembolic complications. Additionally, the emphasis on postoperative surveillance, such as surveillance DSA as proposed by Scullen et al. [19], demonstrates the importance of continuous monitoring in ensuring long-term success and safety.

A careful analysis of preoperative radiological anatomy is recommended to identify the segments of the vertebral artery, as well as the transverse process, the presence of a median vascular loop in the V3 segment, the size and position of the artery in the foramen of the transverse process, and the presence of vertebral body bone erosion due to a vascular loop in the transverse segment. Evaluating collateral vessels makes it possible to anticipate any vascular-related surgical difficulties [5, 27, 38, 39]. Our patient's case report is an isolated and exceptional event; the complication occurred late, and its presumptive diagnosis was based on the presence of nonspecific symptoms in the upper limbs, which were not thoroughly analyzed. Therefore, if a traumatic injury to the AV is suspected or the patient presents mild clinical deterioration, an angiogram should be performed, and treatment should not be delayed.

Endonasal endoscopic approach to the craniocervical junction

Pathologies at the craniovertebral junction are challenging to treat for surgeons due to the precarious anatomical location of these lesions. Surgeons must thoroughly understand the anatomical nuances and biomechanical properties of this area for proper approach selection to minimize morbidity. Over the last two decades, advancements in microsurgical instruments, neuronavigation, and endoscopic techniques have allowed for enhanced endoscopic endonasal approaches to the craniovertebral junction. Thus, the endoscopic endonasal approach has become a viable option for treating lesions such as basilar invagination, cervical rheumatoid pannus, os odontoideum, and upper cervical deformities or tumors, like the presented case [40].

The expansion of the endoscopic endonasal approach toward the cranio-vertebral junction represents its extreme inferior extension on the sagittal plane. It allows to access this relevant region directly, where osteoarticular structures, such as dens and their ligaments, the arch of C1, and the condyles protect the medulla and the upper part of the cervical spine [41]. This approach has been initially proposed by Kassam et al. in 2005 based on several anatomical studies, for odontoidectomy in the case of ventral irreducible brainstem compression, such as basilar invagination [42]. Over the last decade, this route has been adopted not only for degenerative or congenital cranio-vertebral diseases but also for tumors of this region such as chondrosarcomas, chordomas, or meningiomas [8-10]. Nowadays, many routes rather than the endoscopic endonasal vidian nerve approach are available to treat these diseases, such as the transoral, the transcervical, and the transcranial with or without condylectomy [41].

Antibiotic prophylaxis with fourth-generation cephalosporins is performed, with the patient in a supine position, with the head in a neutral position. Endonasal endoscopy is performed with two hands for a greater field of surgical visualization, an endoscope with a 0º lens is introduced into the right nostril, and the surgical instruments are inserted into the left nostril. It is recommended that this procedure be performed in conjunction with an otolaryngologist specializing in ear surgery. The middle turbinate is removed, the sphenoidotomies are performed and the anatomical landmarks are identified: carotid canals, medial pterygoid plates, the pterygoid canal, vidian nerve (nerve of the pterygoid canal), and the fossa of Rosenmüller. An inverted U-shaped flap in the nasopharyngeal mucosa is created from the level of the sphenoidotomy rostrally to the soft palate caudally. The flap is reflected down to the soft palate to expose the sphenoid floor. The floor of the sphenoid sinus is opened and C1 is exposed after dissecting the paraspinal muscles; the anterior arch of C1 as well as part of the lower portion of the clivus is removed in order to expose the odontoid better and perform its resection. By using fibrin glue, the risk of cerebrospinal fluid fistula is considerably reduced. When adequate exposure is made, the risk of vascular injury is very low [10, 35, 37].

Posterior occipito-cervical arthrodesis

It is generally performed with the support of sensory and motor evoked potentials, in a prone position on a Jackson/Misuho table, maintaining cervical alignment and a neutral occipital position, with an incision in the midline, from the inion to the lowest level to be instrumented (in our case, lateral masses of C3, C4 and C5), the fascia is preserved near the inion to facilitate closure and coverage with muscle, the posterior arch of C1 is exposed and dissection is made with Penfield 1; this reduces the risk of vascular injury. Decompression is performed (cervical, occipital or occipito-cervical) and occipital fixation with screws and plate is performed. There are other methods such as fixation with screws towards the occipital condyles wherein it is recommended to have monitoring of the hypoglossal nerve; the alternative is occipital fixation with cables. The plate should be placed below the inion and above the foramen magnum, and as in our case, cervical fixation is usually performed below the lateral masses of C2; although it can also be instrumented, in our case, due to the lytic lesion and previous corpectomy, the procedure was performed below C2 as usual. Complications have been reported to range between 12-30%, including lesions of the vertebral artery, for which an analysis of preoperative imaging studies should be performed, taking into account anatomical variants of the artery, dominant vertebral artery, and/or hypoplastic, tortuous, pronounced loops, etc. [22, 43-46]. Winegar et al. [47] have done a literature search including 34 studies and 799 adult patients. Surgical adverse events were recorded in 24%; postoperative adverse events were recorded in 16.4% of cases (a total of 40.4%). Surgical adverse events included instrumentation misplacement, vascular injury, significant blood loss, thecal sac injury and subsequent CSF leak, and complications from anesthesia. Postoperative adverse events included neurological deterioration, wound complications such as infection and dehiscence, increased pain, cardiac or respiratory events, and pseudarthrosis. In that systematic review, instrumentation failure was recorded in 22.3% of the cases. Major potential complications are spinal cord and nerve root injury, cerebellar injury, posterior fossa hematoma, meningitis, vertebral artery injury, and pseudoarthrosis requiring reoperation [48]. Minor complications are wound infection or dehiscence, dural tears, and cerebrospinal fluid leakage [48]. Among minor complications is the patient’s head position in extremes. Swallowing difficulty and dissatisfaction with the postoperative head position are among the minor complications [49]. Bagley et al. [49] have recommended a preoperative halo immobilization to determine whether the patient will be able to tolerate the new head position. Then, the permanent position during surgery is provided accordingly.

Endovascular management of vertebral pseudoaneurysm

There are multiple devices to perform the endovascular approach and treatment of aneurysms and pseudoaneurysms, from flow diverters, coil placement, and mixed techniques for the treatment of aneurysms and pseudoaneurysms [19, 21, 24, 50-56]; in this case, a covered stent was used with successful results. Nowadays, endovascular management for vertebral artery injury is widely facilitated by advances in techniques and devices. In the case of iatrogenic vertebral artery injury, intra-operative or urgent conventional angiography along with emergent bleeding control is recommended. Conventional angiography in vertebral artery injury (VAI) can identify the exact injury mechanism and site, and evaluate the status of bilateral VA and collateral circulation [15]. Actually, there may be a limitation in having a trained endovascular team and equipment as soon as hemodynamic instability is discovered. However, recent trends suggest that the popularization of endovascular treatment for cerebrovascular disease has led to increased availability of experienced interventionists with well-equipped angio-suites or hybrid operating rooms at several institutions, and improved access to angiography with endovascular treatment for vertebral artery injury [57]. Although there is no consensus on the optimal treatment strategy for vertebral artery injury in cervical spine surgeries, a systematic management method has been reported [22,58]. In brief, when possible, direct surgical treatment such as microvascular repair or surgical ligation should be attempted. However, suturing is technically difficult considering the bleeding site and that surgical ligation is required in the presence of posterior circulation collateral flow. Endovascular treatment for vascular injury can be divided into reconstructive and deconstructive techniques with vertebral artery preservation and occlusion, respectively. If sufficient collaterals exist, coil trapping of the vertebral artery is possible. A stent-graft, multiple stents, and a flow-diverting stent can be considered if vessel preservation is necessary [22]. In conclusion, iatrogenic pseudoaneurysms of the vertebral artery during cervical surgery are uncommon but potentially life-threatening. Endovascular treatment options for iatrogenic vertebral artery injury are limited if there is no contralateral arterial patency or an appropriate instrument in the angiosuite.

## Conclusions

Vertebral pathology secondary to multiple myeloma, particularly at the cranio-cervical junction, presents unique clinical challenges due to the complex biomechanics of the region. Our discussion underscores the importance of an individualized approach, often requiring combined interventions to mitigate neurological deficits. The currently preferred intervention, endoscopic endonasal odontoidectomy, offers patients symptomatic relief and decompression, proving its efficacy in the clinical setting. However, the potential risk of vertebral artery injury, especially during posterior surgical approaches, remains a significant concern. This study sheds light on the need for surgeons to be aware of the potential complications, to remain updated with the latest interventions, and to always prioritize the safety and well-being of the patient. Furthermore, it emphasizes the gap in our current literature, especially regarding specific regions, suggesting the need for more comprehensive research.
